# Oxidative Stress and Apoptosis in Benzo[a]pyrene-Induced Neural Tube Defects

**DOI:** 10.1016/j.freeradbiomed.2018.01.004

**Published:** 2018-02-20

**Authors:** Shanshan Lin, Aiguo Ren, Linlin Wang, Yun Huang, Yuanyuan Wang, Caiyun Wang, Nicholas Greene

**Affiliations:** a Institute of Reproductive and Child Health, Ministry of Health Key Laboratory of Reproductive Health, and Department of Epidemiology and Biostatistics, School of Public Health, Peking University Health Center, Peking University, Beijing, China; b Developmental Biology and Cancer Programme, UCL Great Ormond Street Institute of Child Health, University College London, WC1N 1EH, London, United Kingdom

**Keywords:** NTDs, neural tube defects, PAHs, polycyclic aromatic hydrocarbons, BaP, benzo[α] pyrene, ROS, reactive oxygen species, CYP, cytochrome P450, AhR, aryl hydrocarbon receptor, Sod, superoxide dismutase, Gpx, glutathione peroxidase, Cat, catalase, 8-OHdG, 8-hydroxy-2’-deoxyguanosine, PC, protein carbonyl, 8-iso-PGF2α, 8-iso-prostaglandin F2α, TAC, total antioxidant capacity, MDA, malondialdehyde, h_PAHs, high molecular weight PAHs, Neural tube defects, Polycyclic aromatic hydrocarbons, Benzo[a]pyrene, Oxidative stress, Apoptosis, Vitamin E

## Abstract

Neural tube defects (NTDs) are among the most common and severe congenital malformations and result from incomplete closure of the neural tube during early development. Maternal exposure to polycyclic aromatic hydrocarbons (PAHs) has been suggested to be a risk factor for NTDs and previous studies imply that the mechanism underlying the association between PAH exposure and NTDs may involve oxidative stress and apoptosis. The objectives of this study were to investigate whether there is a direct effect of maternal benzo[α] pyrene (BaP) exposure on the closure of the neural tube in mice, and to examine the underlying mechanisms by combining animal experiments and human subject studies. We found that intraperitoneal injection of BaP from embryonic day 7 at a dose of 250 mg kg^-1^ induced NTDs (13.3% frequency) in ICR mice. BaP exposure significantly increased expression of genes associated with oxidative stress, *Cyp1a1*, *Sod1* and *Sod2*, while repressing *Gpx1*. Elevated apoptosis and higher protein expression of cleaved caspase-3 in the neuroepithelium of treated embryos were observed. Pre-treatment with vitamin E, added to food, significantly protected against BaP-induced NTDs (1.4% frequency) (*P* < 0.05). Vitamin E also partly normalized oxidative stress related gene expression and excess apoptosis in BaP-treated embryos. Examination of human neural tissues revealed that increased levels of protein carbonyl and apoptosis were related with maternal exposure to PAHs and the risk of NTDs. Collectively, these results suggest that BaP exposure could induce NTDs and that this may involve increased oxidative stress and apoptosis, while vitamin E may have a protective effect.

## Highlights

• Intraperitoneal BaP injection induced neural tube defects (NTDs) in foetal mice.
• Maternal BaP exposure altered oxidative stress related gene expression and induced apoptosis in foetal mice.
• Vitamin E rescued BaP-induced NTDs in foetal mice may partly realized via its antioxidative and anti-apoptotic properties.
• PAH concentrations were associated with protein carbonyl (PC) levels in human maternal serum.
• Elevated PC and apoptosis in human foetal neural tissues were associated with increased NTD risk.

## Graphical abstract

**Figure:**
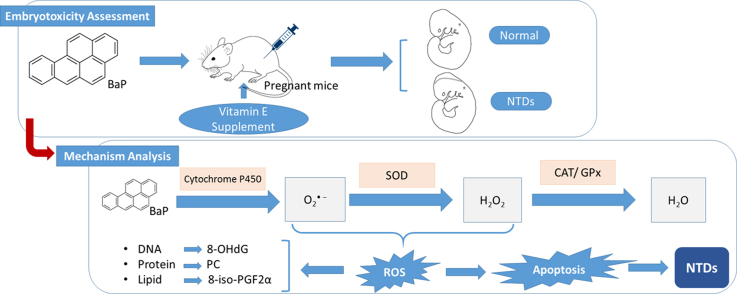

## 1 Introduction

Neural tube defects (NTDs) are a group of common and devastating congenital malformations that arise early in pregnancy due to disturbance of normal neural tube closure. NTDs occur in about one in every 1,000 established pregnancies worldwide [1], and it is estimated that over 323,000 births were affected with NTDs globally in 2001 [2]. The aetiology of NTD is thought to be heterogeneous, including genetic and environmental factors and their interactions [1], [3]. Factors that have been found to be associated with the risk of NTDs include insufficient folate [4] or multivitamin [5] intake, pre-gestational and gestational diabetes [6], pesticides [7] and anti-epileptic drugs [8]. However, the proportion of NTD cases that can be attributed to known risk factors is lower than one-third [9].

Polycyclic aromatic hydrocarbons (PAHs), a class of semi-volatile organic compounds ubiquitously present in the environment, are produced from the incomplete combustion of organic matter. PAHs represent a potential health threat worldwide, and have been associated with a variety of toxic effects, including developmental and immunological disorders, mutagenesis and carcinogenesis [10], [11], [12]. Previous studies have shown that PAHs can interfere with key processes of neuronal development, including migration, differentiation, synaptogenesis, myelination and apoptosis [13]. It is suggested that maternal occupational exposure to PAHs is associated with an increased risk of NTDs in offspring [7], [14], [15]. Human epidemiological studies have found an association between higher concentrations of PAHs in the venous blood of pregnant women and placental tissue with an elevated risk for NTDs, with high molecular weight PAHs (h_PAHs) conferring a higher risk [7], [16]. In addition, we found that higher levels of PAH-DNA adducts in foetal tissues were associated with increased risks of NTDs [17]. Thus, it was hypothesised that exposure to PAHs may be a causal event in the development of NTDs. However, it is not known whether PAHs directly affect mammalian neural tube formation or the mechanism by which they do so.

Following absorption, PAHs undergo intracellular biotransformation to reactive intermediates by cytochrome P450 (CYP) enzymes, leading to production of reactive oxygen species (ROS) [18]. This suggests a possible mechanism by which PAHs could affect neural tube closure as oxidative stress, defined as a disturbance in the balance between the production of ROS and antioxidants [19], has been suggested to contribute to development of some congenital malformations [20]. For example, studies to delineate the mechanism underlying maternal diabetic embryopathy have demonstrated that oxidative stress is a major contributor in NTD formation [21], [22], [23]. Excess apoptosis may be one of the mechanisms by which oxidative stress induces malformations. Apoptosis occurs at various developmental stages as a homeostatic mechanism to maintain cell populations in tissues [24]. During the formation of the neural tube, apoptosis appears to be dispensable; however excessive apoptosis could potentially result in NTDs by causing insufficient cells to be present in the fusing neural folds or by disrupting the physical continuity of the dorsal midline [1], [25]. Excess apoptosis is observed in the neuroepithelium of rodent embryos exposed to high levels of glucose in maternal serum, and deletion of pro-apoptosis kinase genes reduces the incidence of NTDs in these embryos [26], [27].

Interestingly, our previous studies found higher levels of oxidative damage markers in the serum of pregnant women who delivered NTD-affected foetuses than women who delivered healthy newborns [28]. Furthermore, we observed more apoptotic cells in neural tissues of NTD cases than normal foetuses [25]. It is well documented that PAHs could induce oxidative stress and human observational studies suggest an association between PAH exposure and oxidative damage with increased risks of NTDs. However, a direct effect of PAH exposure on neural tube closure has not yet been reported. Therefore, the aim of the present study was to examine the effect of benzo[a]pyrene (BaP), one of the most toxic PAHs, on neural tube closure in mice. Moreover, we asked whether BaP exposure affected oxidative stress status and apoptosis in the embryos and investigated the potential protective effects of vitamin E supplementation. In parallel, levels of redox status, makers of macromolecular oxidative damage and apoptosis were analysed in neural tissues of human foetuses with NTDs.

## 2 Material and methods

### 2.1 Experimental animals

ICR mice of 8-9 weeks old weighing 28 ± 2 g were used in the experiment. Female mice were mated with males overnight and vaginal plugs were examined the following morning. Noon on the day of finding a vaginal plug was considered 0.5 days of embryonic development (E0.5). Pregnant mice were randomly divided into 10 groups. In BaP-treatment groups, mice were given BaP intraperitoneally, dissolved in corn oil, from E6.5 or E7 for four days at doses ranging from 250 to 350 mg kg^-1^. Mice in the vitamin E co-exposure group were fed with chow supplemented with the water-soluble (±)-α-tocopherol succinate form of vitamin E (Sigma) beginning from E0.5 (0.125%, w/w). All mice were maintained on a 12-h light/dark cycle and were allowed free access to food and water. On E10.5, pregnant mice were sacrificed by cervical dislocation and the foetuses were removed by caesarean section. The numbers of implantation sites, living foetuses, and reabsorbed or dead foetuses were recorded. All the live foetuses were carefully inspected for visible external malformations under a dissecting microscope. NTD-affected embryos were classified as showing distinct evidence of failed closure of the neural tube. Embryos for histological analysis were fixed in 4% PFA overnight, then were dehydrated in graded ethanol, embedded in paraffin, and cut into 8-μm sections. After deparaffinization and rehydration, all specimens then underwent hematoxylin-eosin staining using a standard procedure. All neural tube sections were photographed and examined. Other embryos were frozen in -80 °C for further analysis. The study protocol was approved by the Institutional Animal Care and Use Committee of Peking University (certificate no. LA2013-36).

### 2.2 Human subjects and sample collection

The population study design has been described in our previous reports [7]. Briefly, subjects were recruited from an ongoing population-based birth defects surveillance system in Shanxi province of northern China. Cases were foetuses/newborns affected by an NTD; controls were term healthy newborns. Information on sociodemographic characteristics was collected through in-person interviews with the mothers and by viewing medical records. Samples of maternal venous blood were collected at delivery or termination of NTD-affected pregnancies, and were stored at -80 °C. Spinal cord and brain tissues were collected from terminated foetuses following diagnose of spina bifida or cranial NTDs, and from induced foetuses with no congenital malformations. All samples were collected by experienced pathologists and frozen immediately for oxidative stress analysis or fixed in 4% PFA for sectioning. The study protocol was approved by the Institutional Review Board of Peking University, and written informed consent was obtained from all women.

### 2.3 Real-time PCR

RNA was isolated from E10.5 embryos using Trizol (Invitrogen); genomic DNA was removed by DNase I digestion (DNA-free, Ambion) and then reverse-transcribed using random hexamers (Superscript VILO cDNA synthesis kit). The abundance of mRNA of aryl hydrocarbon receptor (*AhR*), *Cyp1a1*, *Cyp1a2*, superoxide dismutase (*Sod1*, *Sod2*), glutathione peroxidase 1 (*Gpx1*) and catalase (*Cat*) were analysed using real-time PCR (iTaq^TM^ Universal SYBR Green Supermix, BioRad) on a 7500 Fast Real Time PCR system (Applied Biosystems), with each sample analysed in triplicate. Primers are listed in Suppl. Table 1. Relative quantification of each gene expression level was normalized according to the *Gapdh* gene expression.

### 2.4 Western blot

Embryos at E10.5 were homogenised in RIPA buffer and Bradford assay was used for protein quantitation. Western blot was performed by conventional methods, with 50 μg of protein run per sample on NuPAGE 4 - 12% Bis-Tris gel (Life technologies) and transfer to PVDF membrane (XCell II Blot Module, Invitrogen). Primary antibodies were rabbit anti-cleaved caspase-3 (1:400, Cell Signaling Technology) and mouse anti-GAPDH (1:50,000, EMD Millipore). After incubation with secondary antibody (1:10,000, DAKO), blots were developed using ECL Prime (GE Healthcare Life Sciences) or ECL Western Blotting Substrate (Promega). Densitometry was performed using ImageJ. Results were normalized to the GAPDH loading control. Independent experiments were carried out three to four times for each sample.

### 2.5 TUNEL assay

Whole mount TUNEL was performed on E9.5 embryos using the Apoptag Peroxidase in situ Apoptosis Detection kit (Millipore) according to the manufacturer’s instruction. Briefly, E9.5 embryos were rehydrated and digested with 10 µg ml^-1^ proteinase K for 4 min, followed by 2 mg ml^-1^ glycine, and then re-fixed in 4% PFA. After fixation, embryos were washed in PBT and incubated in equilibration buffer. Embryos were then incubated with terminal deoxynucleotidyl transferase overnight at 37 °C. Detection was performed using an alkaline phosphatase conjugated anti-digoxigenin antibody (Roche) and NBT/BCIP (Roche) as a substrate. After colour development, embryos were photographed with a DFC490 camera (Leica), and then embedded in gelatin/albumin mixture. Sections of 40-μm thickness were made.

Paraffin slides of neural tissues collected from human foetuses were used for apoptotic cell injury assay with the one step TUNEL kit according to the manufacturer’s instruction (Beyotime Institute of Biotechnology). Briefly, slides were dewaxed with xylene and incubated with proteinase K, followed by TUNEL reaction agents for 1 hr. at 37 °C. Under a microscope (at 400-fold magnification), the cells with green fluorescence were defined as apoptotic cells. Four fields were randomly selected from each section and all the cells were successively counted for each field by an individual who was unaware of case-control status of the slides. The ratio of TUNEL-positive cell number to the total cell number was determined.

### 2.6 Oxidative stress evaluation

The methods used for analysing maternal oxidative damage markers in serum have been described in detail elsewhere [28]. Briefly, 8-hydroxy-2’-deoxyguanosine (8-OHdG) was used as an indicator of oxidative DNA damage and measured with the highly sensitive 8-OHdG check enzyme-linked immunosorbent assay (ELISA) kit (IMKOGHS, Jaica, Japan). Protein oxidation and lipid oxidation were determined by the levels of protein carbonyl (PC) and 8-iso-prostaglandin F2α (8-iso-PGF2α), respectively, with ELISA kits both from Cell Biolabs (San Diego, CA, USA). All samples were loaded in duplicate. The concentration of 8-OHdG was expressed as ng/ml, PC as nmol/mg protein, and 8-iso-PGF2α as pg/ml. Antioxidant indicators and oxidative damage markers in foetal neural tissues were determined according to the kit specifications (Nanjing Jiancheng Bioengineering Institute, Nanjing, China). Activity levels of SOD and GPx, total antioxidant capacity (TAC) were used as antioxidant indicators, and content of malondialdehyde (MDA) and PC were used as lipid and protein oxidation respectively.

### 2.7 PAHs analysis

The detailed procedures for PAHs analysis have been described in our previous studies [29]. Briefly, an agilent 7890A-5975C gas chromatograph and mass spectrometer equipped with a HP-5MS capillary column (30 m × 0.25 mm × 0.25 μm) was used to determine the concentration of a total of twenty-seven parent PAHs. Two procedure blanks and a reagent blank were included for each batch. PAH concentration was expressed on a lipid weight basis and reported as ng/g lipid. H_PAHs are the sum of high molecular weight PAHs with four or five benzene rings including pyrene, benz[a]anthracene, chrysene, benzo[b]fluoranthene, benzo[k]fluoranthene and benzo[a]pyrene.

### 2.8 Statistical analyses

The rates of resorption, growth retardation and malformation were calculated as a percentage of the total number of implantations and analysed by Pearson’s χ^2^ test. The abundance of mRNA and protein were expressed as mean ± SE (SD) and were analysed by one-way analysis of variance (ANOVA) followed by LSD (equal variances assumed) or Dunnett's T3 (equal variances not assumed). In human study, differences in proportions of population characteristics between groups were examined with Pearson’s χ^2^ test. Mann-Whitney test was used for comparisons of oxidative damage markers in maternal serum and foetal neural tissues by levels of h_PAHs in maternal serum, with the median of the controls used as the cut-off value. The levels of apoptosis and oxidative damage markers in neural tissue were expressed as median ± range, and compared between NTD cases and controls using Mann-Whitney test. A two-tail *P* value of < 0.05 was considered statistically significant. Statistical analyses were conducted using SPSS 23.0.

## 3 Results

### 3.1 Effects of BaP on neural tube closure in foetal mice

**Fig. 1:**
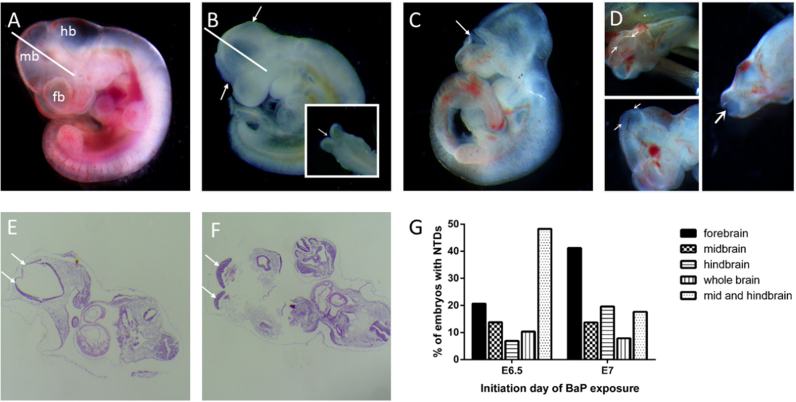
**NTDs in E10.5 mouse embryos exposed to BaP** (A) Control E10.5 embryo; (B-D) E10.5 embryos exposed to BaP with NTDs; (B) Embryo with closure failure in the brain (arrow); (C) Embryo with unfused neural folds resulting in anencephaly (arrow); (D) Embryo with exencephaly near the midbrain (arrow); (E and F) H&E staining of the control and NTD embryos, closed neural tube (E) and open neural plate (F) are indicated by arrows, sectioned along the plane indicated by the white line in A and B, respectively; (G) NTD subtype distribution of E10.5 mouse embryos exposed to BaP. Embryos treated with BaP from E6.5 were found mainly with unfused neural folds at mid and hindbrain, while embryos in E7 exhibited anencephaly with neural folds unfused at forebrain. fb, forebrain; mb, midbrain; hb, hindbrain.

In order to test whether BaP could induce NTDs in a murine model, pregnant mice were intraperitoneally injected with various doses of BaP beginning from E6.5 or E7. On E10.5 when the neural tube would have closed in normal conditions, embryos were isolated and observed under a dissecting microscope. With increasing BaP dose, both the rates of absorbed or dead embryos and the rate of growth retardation increased, with the exception of the group treated with a dose of 200 mg kg^-1^ BaP (Table 1). In the four groups treated from E6.5, NTD rates increased with BaP dose (*P* for trend < 0.05). The group treated with 250 mg kg^–1^ BaP from E7 had the highest rate of NTDs (13.3%), which was approximately 5-fold higher than that of the control group (*P* < 0.01), and showed a relatively high proportion of live embryos (82.8%). Open regions of neural folds were not confined to a specific of the cranial region. The subtypes of NTDs were significantly different in E6.5 and E7 BaP treated groups (*P* < 0.05) (Fig. 1G), with a greater proportion of mid-hindbrain defects among the embryos treated at E6.5, while most of the NTDs induced by BaP from E7 appeared to result from closure failure in the forebrain (Fig. 1B-D). In addition, BaP treatment significantly affected embryo growth as shown by significantly shorter head lengths and crown-rump lengths in groups treated with BaP than the control group (*P* < 0.05) (Suppl. Fig. 1). Other observed malformations included microcephaly, cardiac abnormalities, and curved or short tail. Typical transverse section images of completely closed neural tube of a control embryo and unclosed neural tube of a BaP exposed embryo are shown in Fig. 1E and F, respectively.

**Table 1:** Embryotoxicity of BaP and the effect of vitamin E in ICR mice.

Day	Dose	Litters	Embryos	Death	Resorption	Growth retardation	Cephalic NTDs	All NTDs	Other malformations
	mg kg^-1^	n	n	n (%)	n (%)	n (%)	n (%)	n (%)	n (%)
Blank control		18	226	1 (0.4)	8 (3.5)	7 (3.1)	3 (1.3)	6 (2.7)	2 (0.9)
E6.5	Corn oil	11	136	1 (0.7)	2 (1.5)	2 (1.5)	2 (1.5)	2 (1.5)	2 (1.5)
E6.5	200	11	136	18 (13.2)^a^	30 (22.1)^a^	32 (23.5)^a^	8 (5.9)^a^	11 (8.1)^a^	78 (57.4)^a^
E6.5	250	15	203	21 (10.4)^a^	35 (17.2)^a^	26 (12.8)^a^	7 (3.4)^a^	14 (6.9)^a^	74 (36.5)^a^
E6.5	300	12	163	31 (19.0)^a^	49 (30.1)^a^	61 (37.4)^a^	14 (8.6)^a^	18 (11.0)^a^	78 (47.9)^a^
E7	Corn oil	12	151	0	9 (6.0)	1 (0.7)	3 (2.0)	3 (2.0)	3 (2.0)
E7	250+VE	11	144	1 (0.7)	13 (9.0)	18 (12.5)	**1 (0.7)**	**2 (1.4)**	68 (47.2)
E7	250	24	331	16 (4.8)^b^^c^	43 (13.0)^b^	54 (16.3)^b^	**31 (9.4)**^b^^c^	**44 (13.3)**^b^^c^	120 (36.3)^b^
E7	300	14	177	9 (5.1)^b^	23 (13.0)^b^	26 (14.7)^b^	12 (6.8)^b^	15 (8.5)^b^	79 (44.6)^b^
E7	350	10	136	18 (13.2)^b^	4 (2.9)	33 (24.3)^b^	8 (5.9)^b^	13 (9.6)^b^	69 (50.7)^b^

a *P* < 0.05, compared with E6.5 corn oil;

b *P* < 0.05, compared with E7 corn oil;

c *P* < 0.05, compared with E7 250+VE. VE, vitamin E.

### 3.2 Effect of maternal vitamin E supplementation on NTDs induced by BaP

We hypothesised that BaP could act to induce oxidative stress so we tested whether treatment with the antioxidant vitamin E alleviates the effect of BaP on neural tube closure. Pregnant mice treated with BaP from E7 with 250 mg kg^-1^ were fed with vitamin E supplemented chow beginning from E0.5. Although vitamin E had no significant effects on foetal resorption and growth, it significantly attenuated BaP induced foetal lethality and NTDs (Table 1). Compared with the BaP treated group (E7 250 mg kg^–1^), NTD rate of the vitamin E supplemented group (1.4%) was decreased to a level close to that of the control group.

### 3.3 Effect of BaP exposure and vitamin E supplementation on oxidative stress related gene expression

**Fig. 2:**
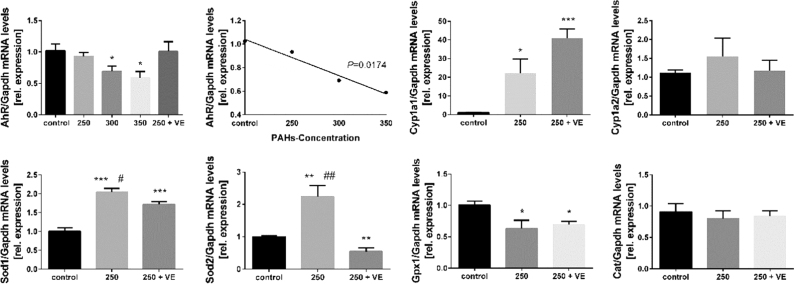
**Relative expression of mRNA of E10.5 embryos exposed to BaP and co-exposed to vitamin E by real-time PCR.** Data for *AhR*, *Cyp1a1*, *Cyp1a2*, *Sod1*, *Sod2*, *Cat* and *Gpx1* were normalized by *Gapdh* for each sample (mean ± SE; n = 4-8). ^*^P < 0.05 or ^**^P < 0.01 or ^***^P < 0.001 vs control; ^#^*P* < 0.05 or ^##^*P* < 0.01 or ^###^*P* < 0.001 vs vitamin E supplemented. VE, vitamin E.

We further hypothesised that vitamin E may prevent BaP-induced NTDs by acting as an antioxidant. To test this hypothesis, we analysed the mRNA levels of several enzymes, including *Cyp1a*, *Sod1*, *Sod2*, *Cat* and *Gpx1*, whose expression is associated with oxidative stress. We also analysed *AhR,* encoding the Aryl Hydrocarbon Receptor which is thought to mediate transcriptional signals in response to environmental toxins such as PAHs. The expression of *AhR* in the BaP-treated group was lower than that in controls (Fig. 2) and a linear correlation between the levels of *AhR* mRNA and BaP dose was observed. Notably, vitamin E treatment normalised *AhR* expression level. Expression of the oxidative stress responsive genes *Cyp1a1* and *Cyp1a2* was increased in BaP-treated embryos, with the effect on *Cyp1a1* being more pronounced, as shown by almost 20-fold higher expression in BaP exposed embryos than in the control group (*P* < 0.05). Unexpectedly, in the vitamin E co-exposure group, *Cyp1a1* mRNA showed a significant increase compared to the BaP-treated group, while *Cyp1a2* was not responsive to vitamin E.

Analysis of expression of mRNA encoding antioxidant enzymes, *Sod1* and *Sod2*, showed that the expression of both genes increased in the BaP-treated group by nearly 2-fold. When vitamin E was co-administered, the induced expression of *Sod1* and *Sod2* was normalised. In contrast to *Sod* genes, BaP-treated embryos showed a significantly decreased level of *Gpx1*, and co-administration with vitamin E failed to restore the level to that of the control group. *Cat* gene expression was not responsive to BaP or vitamin E.

### 3.4 The role of apoptosis in BaP induced NTDs

**Fig. 3:**
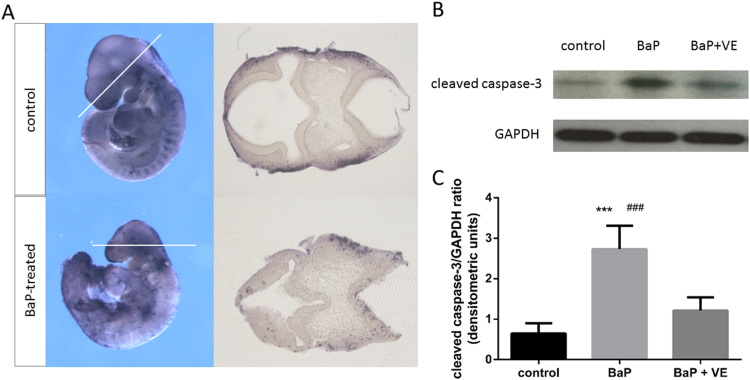
**The effects of BaP and vitamin E on apoptosis levels in E9.5 and E10.5 embryos.** (A) Whole mount in situ TUNEL staining was performed on E9.5 embryos to detect BaP-induced apoptosis (n = 6). The dashed lines on the whole mount panels indicate the orientation of respective sections. (B) Representative western blot images of cleaved caspase-3 and GAPDH expressions. (C) Expression of cleaved caspase-3 in E10.5 embryos was normalized against GAPDH and represented as mean ± SD to that of control (n = 6). ^***^P < 0.001 vs control; ^###^*P* < 0.001 vs vitamin E supplemented.VE, vitamin E.

The smaller size and head length of BaP-treated embryos (Suppl. Fig. 1) suggested a possible effect of BaP on cell proliferation or death. Given the association of oxidative stress with cell death, we examined whether apoptosis was altered in BaP-exposed embryos by whole mount TUNEL assay (Fig. 3A). Apoptotic cells were detectable along the dorsal midline of the whole brain of both control embryos and BaP-treated embryos. However, transverse sectioning showed more apoptotic cells at defective sites of BaP-treated embryos, and apoptosis in the hindbrain region was most obvious, while neuroepithelial apoptosis was relatively low in control embryos.

Cleaved caspase-3 is considered as the end point of caspase activation and provides a measure of apoptosis. We analysed cleaved caspase-3 levels by western blot of samples derived from E10.5 embryos. The protein abundance of cleaved caspase-3 in BaP-treated embryos was much higher than that in the control group, and co-exposure to vitamin E attenuated the effect of BaP (Fig. 3B and C).

### 3.5 PAH exposure, oxidative stress, apoptosis and NTDs in human subjects

Having found that BaP exposure can directly cause NTDs in mice and that this may involve induction of oxidative stress and excess apoptosis, we asked whether there was evidence for the presence of these features in human NTDs, which would suggest a potential contribution of PAH exposure to NTD aetiology. The characteristics of human NTD cases and controls are summarized in Suppl. Table 2. We determined the concentration of PAHs and oxidative damage markers in maternal serum of these subjects. A subgroup with available foetal neural tissues was further used to evaluate the possible association of oxidative status and apoptosis levels in neural tissues with the risk of NTDs. Detailed information on the demographic characteristics of this sub-population is shown in Suppl. Table 3. No significant differences were observed between cases and controls with regard to maternal characteristics, including maternal age and foetal gestational age.

**Fig. 4:**
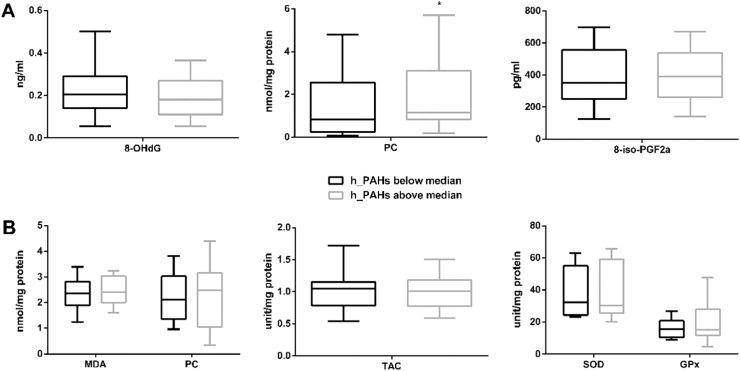
**Oxidative stress and antioxidative stress markers by levels of h_PAHs in maternal serum.** (A) Levels of maternal markers of macromolecular oxidative damage in maternal serum by h_PAHs in maternal serum (n = 230). The maternal PC level of the h-PAHs above the median group was significantly higher than that of the below median group (*P* = 0.019). (B) Markers of redox status and markers of macromolecular oxidative damage in foetal neural tissues by h_PAHs in maternal serum (n = 23). Serum h_PAHs concentration, dichotomized with the median of PAH in control group as the cut-off. ^*^P < 0.05, compared with control group.

A significantly higher level of PC was observed in serum of women whose serum h_PAHs concentration was above the median concentration of the controls when compared to those below the median (Fig. 4), while the concentration of 8-OHdG and 8-iso-PGF2α levels in maternal serum did not differ significantly with PAH levels. We also asked whether the MDA, TAC, SOD or GPx activity in foetal neural tissue correlated with maternal serum h_PAH concentration. However, we did not observe significant between the ‘high’ and ‘low’ maternal h_PAH groups.

**Fig. 5:**
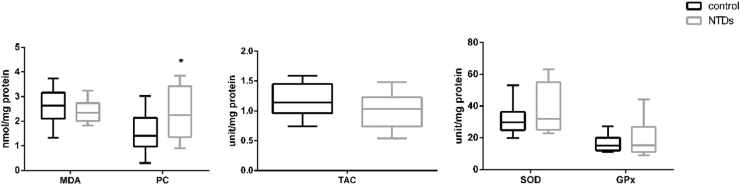
**Association between oxidative status in foetal neural tissue and NTDs.** Comparison of redox status (TAC, SOD and GPx) and makers of macromolecular oxidative damage (PC and MDA) in foetal neural tissues between NTDs (n = 27) and controls (n = 10). ^*^P < 0.05, compared with control group.

Next, we compared levels of oxidative stress markers in neural tissue from foetuses with NTDs and controls (no NTDs). MDA levels did not differ but the median concentration of PC in neural tissues was significantly higher in the case group (2.25 nmol/mg protein) than that in the control group (1.41 nmol/mg protein) (*P* = 0.037) (Fig. 5), suggesting the presence of oxidative stress in NTD neural tissues. The activities of SOD and GPx tended to be higher in case group, and the level of TAC tended to be lower in the case group; however, the differences were not statistically significant.

**Fig. 6:**
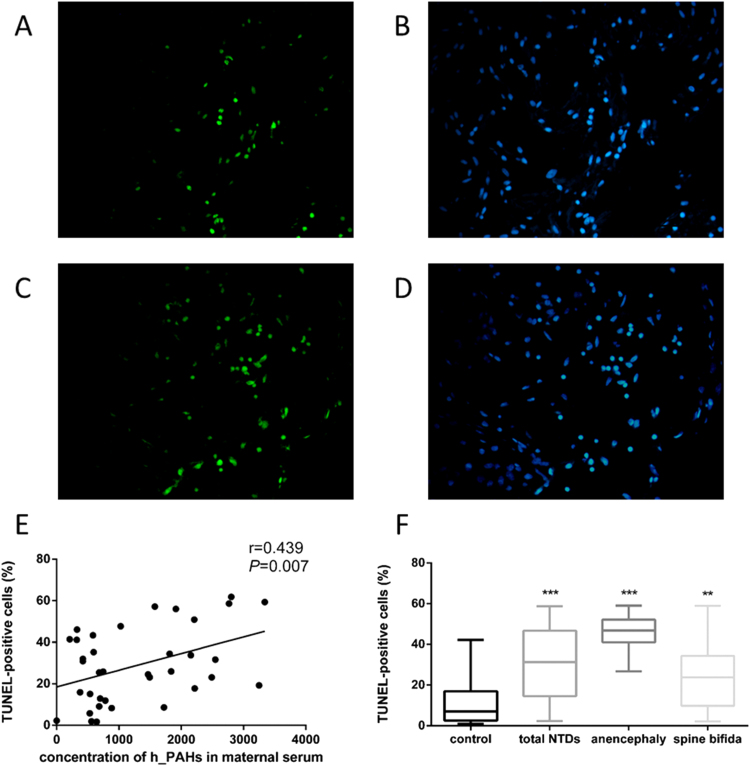
**Apoptosis in foetal neural tissue detected by TUNEL.** (A-D) Representative images of TUNEL-stained neural tissue from a NTD case (C and D) and a control (A and B). TUNEL-positive nuclei were in green fluorescent colour and DAPI staining was performed to visualize the total cell number (blue). (E and F) Apoptosis rate was calculated as percent of TUNEL-positive cells out of total cells (median ± range). (E) Correlation between apoptosis level in foetal neural tissue and h_PAHs concentration in maternal serum (n = 39). (F) Comparison of apoptosis rate in foetal neural tissue between NTD-affected foetuses (n =39) and controls (n = 18). Data were presented as box plots, where the boxed represent the 25h to 75th percentiles, the lines within the boxes represent the median, and the lines outside the boxes represent the 10th and 90th percentiles. ^**^P < 0.01, ^***^P < 0.001, compared with control group.

Having detected a higher abundance of PC in neural tissues of NTD foetuses, we tested whether apoptosis may also occur at a higher level by performing TUNEL staining on tissue sections. As shown in Fig. 6A-D, more TUNEL-positive cells were observed in case neural tissues than that in control neural tissues. The median percentage of TUNEL-positive cells in foetal neural tissue was 30.92% in cases, significantly higher than that of controls, which was 7.06% (Fig. 6F). Subtypes of NTDs, namely anencephaly and spina bifida, also showed higher percentages of TUNEL-positive cells than controls (46.84% for anencephaly; 23.79% for spina bifida). The percentage of TUNEL-positive cells in foetal neural tissues was positively correlated to the concentration of PAHs in maternal serum (Fig. 6E).

## 4 Discussion

PAHs are ubiquitous environmental pollutants. Our [7], [16], [17] and other [14] epidemiological studies have suggested that maternal exposure to PAHs is associated with an elevated risk of NTDs in the offspring. However, evidence from animal experiments is limited. It has been shown that BaP can interfere with anterior neuropore closure in mouse whole embryo culture [30], [31]. However, maternal and placental effects could not be evaluated in this model. In another study, benzo(a)pyrene-7,8-dihydrodiol-9,10-epoxide, an important metabolic derivative of BaP, was injected directly into mouse embryos on E10 [32]. However, neural tube closure is almost finished in mice by E10 [1], such that cranial closure could not be evaluated, and exposure through direct injection also bypasses maternal metabolism. In this context, we focused on deciphering whether PAHs could induce NTDs through maternal exposure during early organogenesis. We found that intraperitoneal treatment with BaP on E7 can result in significantly increased incidence of NTDs. This mouse model may be of significance in elucidating the epidemiological association between maternal PAH exposure and NTDs.

Oxidative stress has been implicated in the aetiology of congenital anomalies caused by maternal diabetes, valproic acid, alcohol intake and several environmental toxins [8], [33], [34], [35], [36]. After entering the body, BaP binds with AhR, activates the expression of CYP genes, and is metabolized by CYP enzymes to epoxides, which are further hydrated to various dihydrodiols by epoxide hydrolase, resulting in oxidative stress [18]. AhR is a member of the transcription factor, which is well-known to mediate the toxicological response of environmental contaminants such as PAHs. Recently, AhR is proposed as a potent suppressor of oxidative stress [37]. The decreased expression of *AhR* observed in present study may imply the repressed antioxidant capacity of embryos after BaP treatment. Expression of *Cyp1a1* and *Cyp1a2* were selected as indicators for activation of oxidative stress [38], [39]. A dramatically increased level of *Cyp1a1* mRNA was observed in the BaP-treatment group, which indicates possible activation of oxidative stress induced by BaP. However, the expression of *Cyp1a2* was not altered after BaP treatment. The differential induction of *Cyp1a1* and *Cyp1a2* was also found in other studies on BaP [40]. A possible explanation for this is that *Cyp1a2* is constitutively expressed at higher levels mainly in liver, while *Cyp1a1* is primarily extrahepatically expressed, with brains as one of the main organs [41], [42], [43]. Thus, our findings are supported by previous studies and suggest that *Cyp1a1* is of more significance in local metabolism of BaP in neural tissues during early development in mice.

Stimulation of the expression of genes encoding oxidative stress-scavenging enzymes has been proposed to act as a mechanism of defence against damage induced by oxidative stress during diabetic pregnancy [44], [45]. SODs represent a family of cellular enzymes involved in converting superoxide into peroxide, which can then be converted into water by CAT and GPx. We observed in present animal study that the levels of *Sods* were elevated in the BaP-treatment group, which has also been reported in mice and rats treated with PCBs, Aroclor 1254 and tetrachlorobiphenyls [46], [47], [48], [49]. However, no increase was seen in the expressions of *Gpx1* and *Cat* after BaP exposure. The absence of the up-regulation of these two antioxidant enzymes was also found in studies done with scallops of *Chlamys farreri* [50], scallops N. nodosus [51], and roach (Rutilus rutilus) [52]. It was demonstrated in cultured mouse embryos that SODs can significantly reduce the incidence of NTDs induced by BaP and ethanol [30], [53]. In present study, the elevated *Sods*, however, were accompanied with an increased incidence of NTDs after BaP exposure. One possible explanation is that although the expression of Sods increased, the repressed *Cat* and *Gpx1* expression after BaP exposure failed to cooperate with *Sods* to detoxify hydrogen peroxide to water, which might favour the accumulation of H_2_O_2_. It was proposed that it is the balance in the activity of the SOD to GPx plus CAT ratio (SOD/(GPX plus CAT)) that is an important determinant in the antioxidant defence system against pathologies [54], [55]. An elevation in the ratio of SODs activity to GPx1/CAT activity was found to be correlated with increased lipid damage and cellular senescence and/or cell death [54], [56], [57]. Our present data are consistent with these studies, supporting the idea that the balance in the antioxidant defence system is of more significance for oxidative stress resistance under stressed conditions.

To further explore the role of oxidative stress in BaP induced NTDs, we co-administered BaP with vitamin E, which has been shown to protect against oxidative stress related BaP metabolism [58], [59], [60]. As expected, the proportion of NTD affected embryos in the vitamin E-supplemented group was considerably lower than that in the BaP-treated group. Pre-treatment with vitamin E attenuated the elevated expressions of *Sod1* and *Sod2* induced by BaP as well. The same regulation effect of vitamin E on *Sods* was also found in animal studies treated with deltamethrin and PCB126 [61], [62]. Unexpectedly, we found that co-administration with vitamin E enhanced the level of *Cyp1a1* about 2-fold compared with the BaP-treatment group. This seemingly conflicting inductive effect of vitamin E on the expression of *Cyp1a1* has been previously reported in oestrogen-induced mammary tumorigenesis [63]. Based on the data from our study, one possible explanation is that vitamin E alleviated the inhibition of BaP on *AhR*, the activation of which could up-regulate *Cyp1a* expression. Furthermore, CYP genes are not only regulated by AhR, but also by other nuclear receptors, including Pregnane X Receptor, which also responds to modulation by vitamin E [64]. Another explanation may be that apart from its antioxidant activity, vitamin E as a lipid-soluble antioxidant, follows the metabolic pathway of dietary lipid in the body, which also involves the activation of CYP enzymes. It is suggested in recent studies that the role of *Cyp1* induction is required for rapid clearance of BaP [65] and the inhibition of *Cyp1* could amplify the carcinogenic and teratogenic effects of PAHs [66], [67]. When *Cyp1a* was inhibited, the rate of PAHs metabolism decreased, extending the half-life of PAHs and allowing parent PAHs to persist longer as evidenced by the increased toxicity of PAHs after *Cyp1a* knockdown or inhibition [68], [69], [70]. This may explain the decreased rate of NTDs in vitamin E supplemented group accompanied with induction of Cyp1a, indicating the protective role of vitamin E may be related with facilitating the clearance of BaP.

Levels of redox status, makers of macromolecular and oxidative damage were also analysed in human tissues to explore whether similar mechanisms were included in the aetiology of human NTDs. And we found increased levels of PC in maternal serum and foetal neural tissues in the group with higher PAH exposure, indicating a potential contribution of maternal PAH exposure to oxidative stress in both pregnant women and foetuses. In addition, the concentration of PC in NTD foetal neural tissues was significantly higher than that of controls. PC is one of the most widely used oxidative markers and the accumulation of PC has been observed in a NTD mouse model induced by maternal diabetes [71], [72], which is in line with our finding. However, unlike in BaP-treated mice, where expression of *Sods* was increased and *Gpx1* decreased, we did not observe a significant change in SODs and GPx activity in human tissue with exposure to higher PAH or in NTDs. This discrepancy may be due to the short-term, more acute treatment in the mouse model in which development stages and the timing of exposure can be strictly controlled. Collection of biological samples from human foetuses is difficult and it is not feasible to obtain neural tissue at the stage when the neural tube is closing. Therefore, the observational human study is subject to the influence of many factors as well as a limited sample size for analysis of oxidative stress and redox markers. Future studies should expand the sample size and collect tissue samples as close to the window of neural tube closure as possible.

Growing evidence indicates that oxidative stress can stimulate apoptosis, which may lead to insufficient cell numbers to participate in folding and fusion of neural walls of the neural tube [73], [74], [75]. In *Cited2* mutant embryos, NTDs are associated with excessive neuroepithelial cell death, which leads to insufficient cell numbers to take part in the crucial morphogenetic movements for neural tube closure [76]. Supportively, we observed increased levels of TUNEL-positive cells and cleaved caspase-3 in the neuroepithelium of the mouse brain after exposure to BaP. And we further found that vitamin E could alleviate the increased expression of cleaved caspase-3 induced by BaP, which imply a possible role of oxidative stress in stimulation of apoptosis. This is in consistent with Numakawa’s study which reported pre-treatment with vitamin E could prevented cell death in cultured cortical neurons stimulated with peroxide [77]. To our knowledge, direct studies of apoptosis in human NTDs foetus are very limited. We showed here that more TUNEL-positive cells were observed in the central nervous tissues of foetuses with NTDs than those of controls. Furthermore, we provided additional evidence of a positive correlation between increased apoptosis levels in foetal neural tissues and a higher concentration of PAHs in maternal serum. Taken together, these findings suggest a role of apoptosis in NTDs induced by BaP, and apoptosis may be activated through the pathway mediated by oxidative stress. However, one limitation is that human tissues were mostly collected at second trimester, while the closure of neural tube completes at the first month of pregnancy. This drawback could not be eliminated with routine case-control design, as the neural tissues of foetus can only be sampled at elective termination after a diagnose of NTDs. In addition, when analysing by gestational age in the case or control group separately, we did not found differences in the apoptosis level between gestational age younger or older than 28 weeks, suggesting that gestational age has no impact on apoptosis level in the present study.

In conclusion, to our knowledge this study is the first to explore whether maternal BaP exposure could induce NTDs in embryos and to assess the potential role of oxidative stress and apoptosis in the aetiology of BaP embryotoxicity. Our results demonstrate that maternal exposure to BaP can induce NTDs in the embryo and suggest that oxidative stress and apoptosis may be involved in the formation of NTDs after BaP exposure. In humans, increased levels of makers of macromolecular oxidative damage and apoptosis are associated with NTDs, which may be also associated with maternal exposure to PAHs. We further show that vitamin E treatment significantly reduced the frequency of NTDs, possibly through its anti-oxidation and anti-apoptosis effects, making antioxidants may be good candidates for preventing NTDs resistant to folic acid. However, in consideration of potentially harmful health effects of excess vitamin E, the recommendation of vitamin E supplementation during pregnancy must carefully balance both the risks and benefits.
