# Supplementary material for: Oxidative Stress and Apoptosis in Benzo[a]pyrene-Induced Neural Tube Defects
Source: Free Radic Biol Med. 2018 Feb 20;116:149–58. doi: 10.1016/j.freeradbiomed.2018.01.004 (PMC5821680; doi:10.1016/j.freeradbiomed.2018.01.004)
Supplement: Supplementary file 1 — Supplementary material [file mmc1.docx]

## Supplementary figure and table





**Fig. 1**. **Head and crown-rump lengths of E10.5 mouse embryos exposed to BaP.** Data were shown as mean ± SD, Error bars represent SD. ^a^*P* < 0.05, compared with control; ^b^*P* < 0.05, compared with E6.5 200; ^c^*P* < 0.05, compared with E6.5 250; ^d^*P* < 0.05, compared with E7 250.


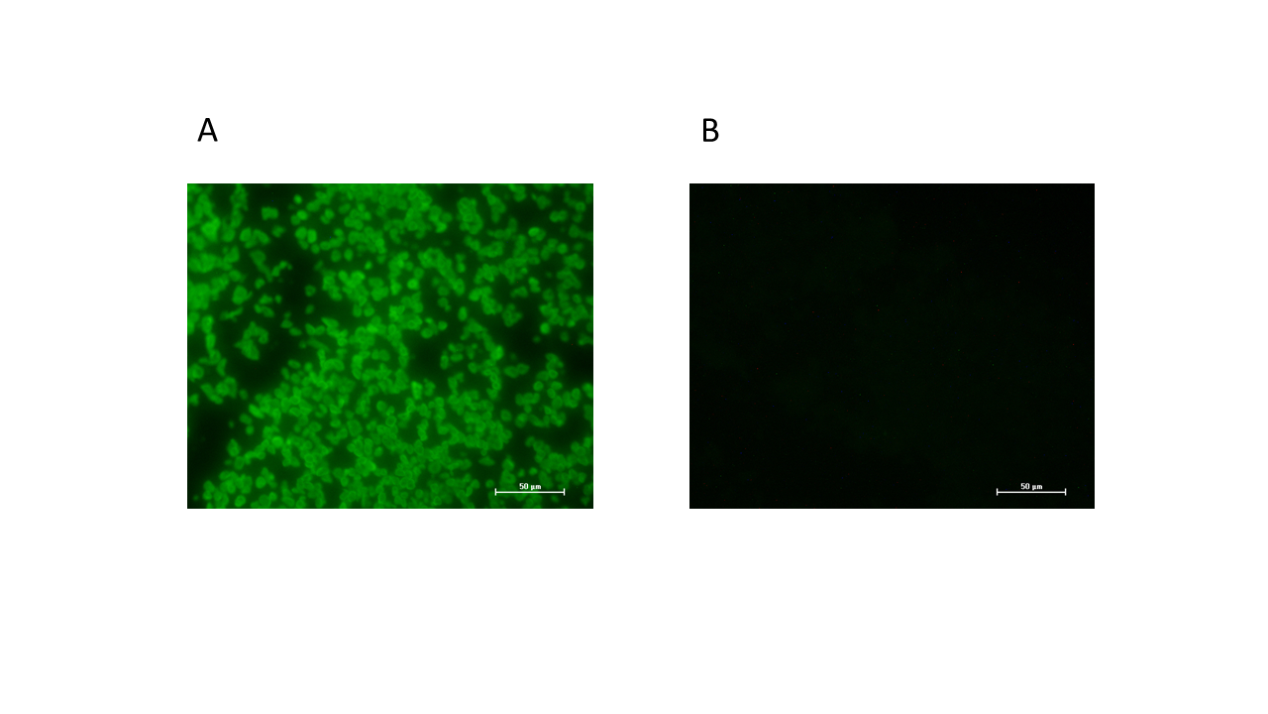


**Fig 2. Positive and negative controls of TUNEL staining.** (A) Positive control, DNA fragmented by DNase Ι. (B) Negative control obtained by omitting TdT enzyme for TUNEL reaction.


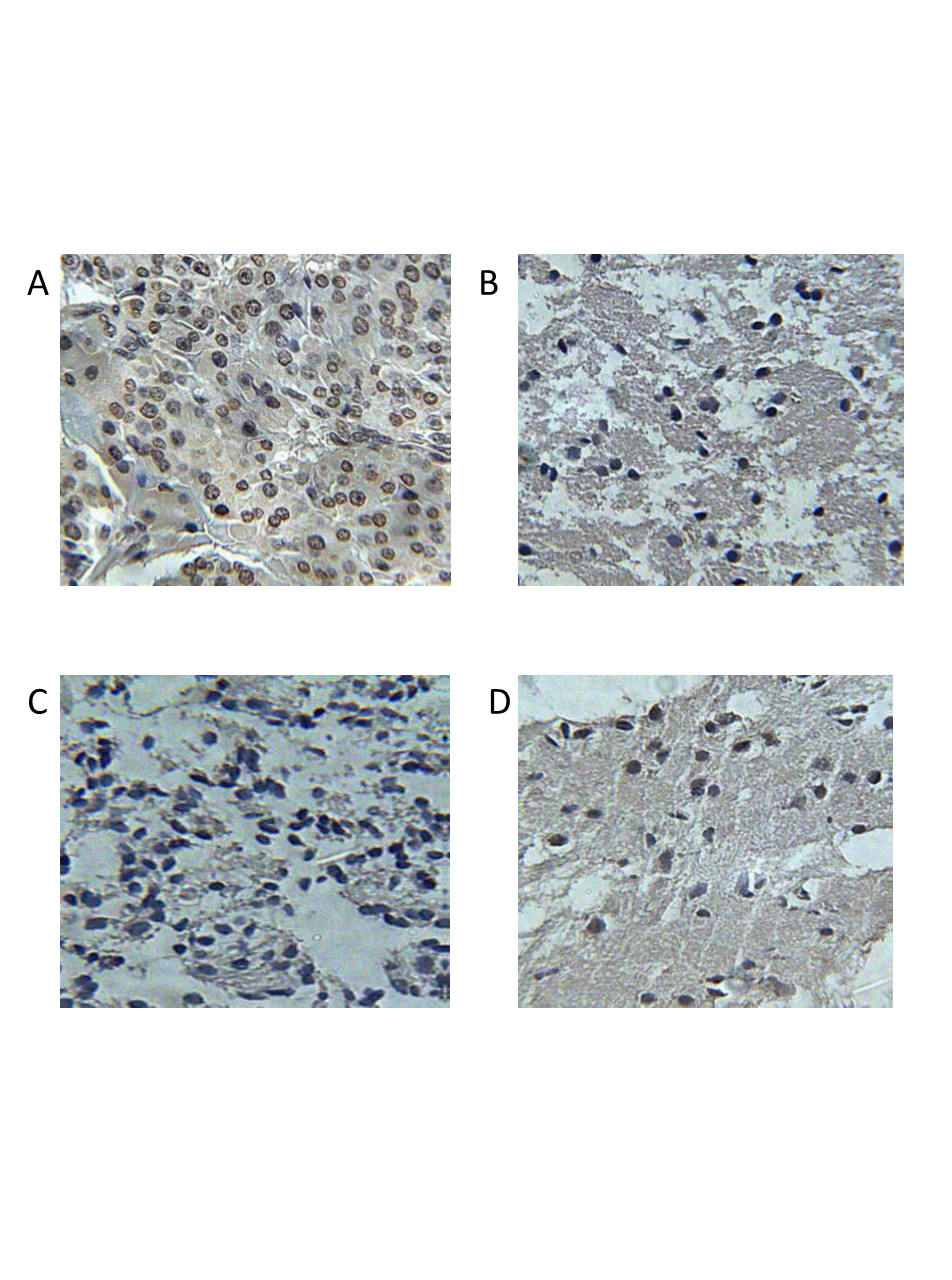


**Fig 3.** **Apoptosis in foetal neural tissue determination by TUNEL assay.** Representative images labeled by TUNEL (brown) and counterstained with hematoxylin (blue). (A) Positive control treated with DNase I. All cells exhibit dark-stained, TUNEL^+^ nuclei. (B) Negative control, obtained by omitting the terminal deoxynucleotidyl transferase enzyme for the TUNEL reaction; all cells are TUNEL^–^. (C) Neural tissue from a control. (D) Neural tissue from a NTD case.

**Table 1. Sequences of primers for real-time PCR**

| Primer | Forward sequence | Reverse sequence |
| --- | --- | --- |
| *AhR* | ACCAGAACTGTGAGGGTTGG | TCTGAGGTGCCTGAACTCCT |
| *Cyp1a1* | TGTCCTCCGTTACCTGCCTA | GTGTCAAACCCAGCTCCAAA |
| *Cyp1a2* | TGGAGCTGGCTTTGACACAG | CGTTAGGCCATGTCACAAGTAGC |
| *Sod1* | CCAGTGCAGGACCTCATTTT | TTGTTTCTCATGGACCACCA |
| *Sod2* | CCGAGGAGAAGTACCACGAG | GCTTGATAGCCTCCAGCAAC |
| *Gpx1* | GTCCACCGTGTATGCCTTCT | TCTGCAGATCGTTCATCTCG |
| *Cat* | CCAGCGACCAGATGAAGCAG | CCACTCTCTCAGGAATCCGC |
| *Gapdh* | ATGACATCAAGAAGGTGGTG | CATACCAGGAAATGAGCTTG |

**Table 2. Characteristics of women who had pregnancies affected by NTD (case) and women who delivered healthy newborns (control) in Shanxi Province, China, 2010-2014**

| Characteristic | |  | Case^a^ (n = 126) | Control^a^ (n = 136) | *P* value^b^ |
| --- | --- | --- | --- | --- | --- |
| Maternal age (y) | | |  |  |  |
|  | < 25 | | 47 (43) | 74 (57) |  |
|  | 25 - 29 | | 30 (27) | 31 (24) |  |
|  | ≥ 30 | | 33 (30) | 24 (19) | 0.050 |
| BMI (kg/m^2^) | | |  |  |  |
|  | < 18.5 | | 11 (9) | 14 (11) |  |
|  | 18.5 - 24.9 | | 64 (54) | 87 (69) |  |
|  | ≥ 25 | | 44 (37) | 25 (20) | 0.012 |
| Maternal education | | |  |  |  |
|  | primary or lower | | 11 (9) | 3 (2) |  |
|  | Junior high | | 90 (72) | 77 (57) |  |
|  | High school or above | | 24 (19) | 56 (41) | <0.001 |
| Occupation | | |  |  |  |
|  | Farmer | | 101 (81) | 96 (71) |  |
|  | Non-farmer | | 23 (19) | 40 (29) | 0.058 |
| Previous birth defects history | | |  |  |  |
|  | Yes | | 8 (6) | 1 (1) |  |
|  | NO | | 117 (94) | 127 (99) | 0.038 |
| Gravidity | | |  |  |  |
|  | 1 | | 53 (42) | 71 (54) |  |
|  | ≥ 2 | | 73 (58) | 61 (46) | 0.078 |
| Parity | | |  |  |  |
|  | 1 | | 98 (82) | 105 (95) |  |
|  | ≥ 2 | | 21 (18) | 6 (5) | 0.007 |
| Periconceptional folate supplementation | | |  |  |  |
|  | Yes | | 60 (48) | 69 (53) |  |
|  | No | | 64 (42) | 61 (47) | 0.449 |
| Fever or flu during early pregnancy | | |  |  |  |
|  | Yes | | 46 (38) | 23 (17) |  |
|  | No | | 76 (62) | 109 (83) | <0.001 |
| Active or passive smoking | | |  |  |  |
|  | Yes | | 71 (62) | 43 (33) |  |
|  | No | | 43 (38) | 87 (67) | <0.001 |
| Drinking | | |  |  |  |
|  | Yes | | 19 (15) | 10 (7) |  |
|  | No | | 107 (85) | 126 (93) | 0.073 |
| Gestational age (weeks) | | |  |  |  |
|  | < 28 | | 71 (58) | 21 (16) |  |
|  | 28 - 36 | | 28 (23) | 11 (9) |  |
|  | >36 | | 23 (19) | 96 (75) | <0.001 |

^a^ Date were presented in number (percentage). Total number may not be equal to the total of cases or controls due to missing or unknown data. ^b^ Cases and controls were compared by Pearson's χ2 test, or Fisher's exact test if any cell expectation was less than 5.

**Table 3. Characteristics of a subgroup of women who had pregnancies affected by NTD (case) and women who delivered healthy newborns (control) in Shanxi Province, China, 2010-2014**

| Characteristic | |  | Case^a^ (n = 47) | Control^a^ (n = 22) | *P* value^b^ |
| --- | --- | --- | --- | --- | --- |
| Maternal age (y) | | |  |  |  |
|  | < 25 | | 18 (42) | 11 (55) |  |
|  | 25 - 29 | | 11 (26) | 4 (20) |  |
|  | ≥ 30 | | 14 (33) | 5 (25) | 0.662 |
| BMI (kg/m^2^) | | |  |  |  |
|  | < 18.5 | | 2 (5) | 2 (10) |  |
|  | 18.5 - 24.9 | | 27 (61) | 10 (50) |  |
|  | ≥ 25 | | 15 (34) | 8 (40) | 0.578 |
| Maternal education | | |  |  |  |
|  | primary or lower | | 7 (15) | 1 (5) |  |
|  | Junior high | | 30 (64) | 12 (55) |  |
|  | High school or above | | 10 (21) | 9 (41) | 0.158 |
| Occupation | | |  |  |  |
|  | Farmer | | 40 (89) | 17 (77) |  |
|  | Non-farmer | | 5 (11) | 5 (23) | 0.277 |
| Previous birth defects history | | |  |  |  |
|  | Yes | | 3 (7) | 0 |  |
|  | NO | | 43 (93) | 22 | 0.546 |
| Gravidity | | |  |  |  |
|  | 1 | | 21 (45) | 12 (55) |  |
|  | ≥ 2 | | 26 (55) | 10 (45) | 0.606 |
| Parity | | |  |  |  |
|  | 1 | | 34 (76) | 13 (72) |  |
|  | ≥ 2 | | 11 (24) | 5 (28) | 0.760 |
| Periconceptional folate supplementation | | |  |  |  |
|  | Yes | | 24 (53) | 9 (41) |  |
|  | No | | 21 (47) | 13 (59) | 0.495 |
| Fever or flu during early pregnancy | | |  |  |  |
|  | Yes | | 17 (39) | 6 (27) |  |
|  | No | | 27 (61) | 16 (73) | 0.421 |
| Active or passive smoking | | |  |  |  |
|  | Yes | | 26 (60) | 9 (43) |  |
|  | No | | 17 (40) | 12 (57) | 0.285 |
| Drinking | | |  |  |  |
|  | Yes | | 10 (21) | 3 (14) |  |
|  | No | | 37 (79) | 19 (86) | 0.528 |
| Gestational age (weeks) | | |  |  |  |
|  | < 28 | | 28 (60) | 10 (45) |  |
|  | 28 - 36 | | 16 (34) | 8 (36) |  |
|  | > 36 | | 3 (4) | 4 (18) | 0.270 |

^a^ Data were presented in number (percentage). Total number may not be equal to the total of cases or controls due to missing or unknown data. ^b^ Cases and controls were compared by Pearson's χ2 test, or Fisher's exact test if any cell expectation was less than 5.

**Table 4. Concentrations of oxidative stress and antioxidative stress markers in maternal serum and foetal neural tissues by levels h_PAHs in maternal serum in Shanxi Province, China, 2010-2014**

|  |  | n | h_PAHs below median | h_PAHs above median | *P* value |
| --- | --- | --- | --- | --- | --- |
| Maternal serum | |  |  |  |  |
|  | 8-OHdG | 230 | 0.20 (0.14-0.28) | 0.18 (0.11-0.27) | 0.270 |
|  | PC | 230 | 0.9 (0.3-3.1) | 1.2 (0.8-3.1) | 0.019 |
|  | 8-iso-PGF2α | 230 | 354.7 (232.8-537.8) | 386.2 (263.1-527.7) | 0.741 |
| Foetal neural tissue | |  |  |  |  |
|  | MDA | 23 | 2.3 (1.9-2.8) | 2.4 (2.0-3.0) | 0.659 |
|  | PC | 23 | 2.1 (1.4-3.0) | 2.5 (1.0-3.2) | 1.000 |
|  | TAC | 23 | 1.1 (0.8-1.2) | 1.0 (0.8-1.2) | 0.850 |
|  | SOD | 23 | 32.4 (24.3-55.1) | 30.4 (25.5-59.2) | 0.950 |
|  | GPx | 23 | 15.7 (10.4-20.8) | 15.1 (11.7-28.2) | 0.450 |

Data were presented in median (interquartile range), Mann-Whitney test was used for comparisons of oxidative damage markers in maternal serum and foetal neural tissues by levels of h_PAHs in maternal serum, with the median of the controls used as the cut-off value. 8-OHdG, ng/ml; PC, nmol/mg protein; 8-iso-PGF2α, pg/ml; MDA, nmol/mg protein; TAC,SOD, GPx, unit/mg protein.
